# Association of diabetes and cancer mortality in American Indians: the Strong Heart Study

**DOI:** 10.1007/s10552-015-0648-7

**Published:** 2015-08-07

**Authors:** Lyle G. Best, Esther García-Esquinas, Jeun-Liang Yeh, Fawn Yeh, Ying Zhang, Elisa T. Lee, Barbara V. Howard, John H. Farley, Thomas K. Welty, Dorothy A. Rhoades, Everett R. Rhoades, Jason G. Umans, Ana Navas-Acien

**Affiliations:** Epidemiology Department, Missouri Breaks Industries Research Inc., 118 S. Willow Str, Timber Lake, 57625 SD USA; Department of Environmental Health Sciences, Johns Hopkins University Bloomberg School of Public Health, Baltimore, MD USA; Department of Preventive Medicine and Public Health, Universidad Autónoma de Madrid, Madrid, Spain; CIBERESP, Madrid, Spain; Center for American Indian Health Research, College of Public Health, University of Oklahoma Health Sciences Center, Oklahoma City, OK USA; MedStar Health Research Institute, Hyattsville, MD USA; Georgetown-Howard Universities Center for Clinical and Translational Science, Washington, DC USA; Division of Gynecology Oncology, Department of Obstetrics and Gynecology, Creighton University School of Medicine at St. Joseph’s Hospital and Medical Center, Phoenix, AZ USA; Stephenson Cancer Center and Department of Medicine, University of Oklahoma Health Sciences Center, Oklahoma City, OK USA; Department of Oncology, Johns Hopkins School of Medicine, Baltimore, MD USA; 1935 118th Ave NW, Watford City, ND 58854 USA

**Keywords:** Diabetes mellitus, Cancer, American Indian

## Abstract

**Purpose:**

The metabolic abnormalities that accompany diabetes mellitus are associated with an increased risk of many cancers. These associations, however, have not been well studied in American Indian populations, which experience a high prevalence of diabetes. The Strong Heart Study is a population-based, prospective cohort study with extensive characterization of diabetes status.

**Methods:**

Among a total cohort of 4,419 participants who were followed for up to 20 years, 430 cancer deaths were identified.

**Results:**

After adjusting for sex, age, education, smoking status, drinking status, and body mass index, participants with diabetes at baseline showed an increased risk of gastric (HR 4.09; 95 % CI 1.42–11.79), hepatocellular (HR 2.94; 95 % CI 1.17–7.40), and prostate cancer mortality (HR 3.10; 95 % CI 1.22–7.94). Further adjustment for arsenic exposure showed a significantly increased risk of all-cause cancer mortality with diabetes (HR 1.27; 95 % CI 1.03–1.58). Insulin resistance among participants without diabetes at baseline was associated with hepatocellular cancer mortality (HR 4.70; 95 % CI 1.55–14.26).

**Conclusions:**

Diabetes mellitus, and/or insulin resistance among those without diabetes, is a risk factor for gastric, hepatocellular, and prostate cancer in these American Indian communities, although relatively small sample size suggests cautious interpretation. Additional research is needed to evaluate the role of diabetes and obesity on cancer incidence in American Indian communities as well as the importance of diabetes prevention and control in reducing the burden of cancer incidence and mortality in the study population.

## Introduction

A growing body of evidence links diabetes and chronic hyperglycemia with the development of several common cancers, including pancreas, liver, colon, and breast cancers [1–5]. In a meta-analysis of 12 studies from Europe, Asia, and the USA, diabetes was also associated with increased all-cause cancer mortality (pooled relative risk 1.16, 95 % CI 1.03–1.30) [6].

Biologic plausibility for a direct causal association between cancer and hyperglycemia was first suggested in the early 1900s by Warburg et al. based on the increased reliance of cancer cells on glucose and aerobic glycolysis (the “Warburg Effect”) [7]. This effect has recently been further corroborated by the detection of increased glucose metabolism in malignant tissue [8] and the demonstration of these metabolic changes in individual cancer cells [9]. The increased availability of glucose may enhance the survival of malignant cells in their early stages or provide a growth advantage during later stages. Insulin, and insulin-like growth factors (IGFs) which are increased during much of the course of type II diabetes [10, 11], could also increase cancer risk.

Cancer has become a leading cause of death among American Indians [12, 13], who have the highest rates of type 2 diabetes in the USA [14–16]. Little is known, however, about the associations between hyperglycemic conditions and cancer among American Indians. In 2003, the Strong Heart Study, a population-based prospective cohort study in American Indian communities of Arizona, Oklahoma, and North and South Dakota, failed to identify an association between diabetes and all-cause cancer mortality over 9 years of follow-up [17]. However, that initial study was limited by a small number of cases of cancer mortality as well as a relatively short period of follow-up. In addition, specific cancers were not evaluated in that report [17].

The Strong Heart Study has continued to follow older American Indians since 1989, contributing important information on the relationship of diabetes and hyperglycemic conditions to complex diseases [18–21]. Although the study was primarily funded to investigate cardiovascular disease and its risk factors, cancer deaths have also been identified as part of the mortality surveillance system. The objective of the current study is to determine the association of diabetes, hyperglycemia, and insulin resistance with cancer mortality among American Indians participating in the Strong Heart Study. Based on the evidence from other populations, we hypothesized that diabetes would be associated with an increase in total cancer mortality, as well as with the following cancer-specific mortalities: gastric, colorectal, breast, renal cell, liver, and pancreas cancer.

## Methods

### Study population

From 1989 to 1991, men and women 45–75 years of age from 13 American Indian communities were invited to participate in the Strong Heart Study. In Arizona and Oklahoma, every eligible person was invited, whereas in North/South Dakota, a cluster sampling technique was used. The baseline participation rate was 62 %, with a final sample of 4,549 participants. All analysis was based on baseline data collected in the first examination of this cohort in 1989–1991. For this study, we excluded participants missing information on diabetes status (*n* = 90), body mass index (BMI) (*n* = 20), smoking status (*n* = 8), alcohol consumption (*n* = 8), and education level (*n* = 4), leaving 4,419 participants for analysis. The SHS protocol was approved by multiple institutional review boards and by the participating communities. All participants provided oral and written informed consent.

### Data collection

Study visits were performed by trained and certified examiners following a standard protocol and included a questionnaire (sociodemographic factors, environmental exposures, and medical history), a physical examination (height, weight, and blood pressure), and bio-specimen collection (blood, urine). Participants were asked to fast for 12 h before blood collection. Plasma glucose was measured by a hexokinase method at MedStar Health Research Institute (MHRI), Hyattsville, MD. A 2-h, 75-g oral glucose tolerance test was performed in participants without diabetes medication. The oral glucose tolerance test was not performed if the participant received renal dialysis (or a renal transplant) or if the fasting glucose level was greater than 225 mg/dL as determined by Acucek II (Baxter Healthcare, Grand Prairie, Texas). HbA1c was measured by a high-performance liquid chromatography method at the laboratory of the National Institute of Diabetes and Digestive and Kidney Diseases Epidemiology and Clinical Research Branch, Phoenix, Arizona. Plasma insulin was measured at MHRI by radioimmunoassay (Linco, St. Louis, Missouri).

### Diabetes definition

Diabetes was defined as a fasting glucose level ≥126 mg/dL or the use of insulin or oral hypoglycemic medication. Those on renal dialysis or with a kidney transplant and that responded positively to the question “Has a medical person ever told you that you had diabetes?” were also classified as having diabetes. The date of diagnosis was either the date of the baseline examination resulting in diagnostic criteria or the self-reported date of diagnosis from the baseline questionnaire. Because type 1 diabetes is rare, we assumed essentially all cases in our study have type 2 diabetes. Impaired fasting glucose (IFG) was defined as fasting glucose ≥100 and <126 mg/dL in the absence of diabetes as defined above. Insulin resistance was estimated in non-diabetic participants using the homeostasis model assessment to quantify insulin resistance (HOMA-IR) = [fasting plasma insulin (mU/L) × fasting plasma glucose (mmol/L)]/22.5. In addition to HOMA-IR, we also evaluated plasma insulin.

### Cancer mortality follow-up

Follow-up for mortality was complete for 99.8 % of the study population. Death certificates were obtained from the State Departments of Health and the underlying cause of death used in analysis. If the death certificate indicated that an autopsy had been performed, the medical examiner’s report was obtained. Death certificate codes were recorded according to the International Classification of Diseases, 9th Revision (ICD-9). In addition to overall cancer, we evaluated the following specific cancers: esophagus and stomach (ICD-9 150–151), colon and rectum (153–154), liver and intrahepatic bile ducts (157) (referred to from now on as liver cancer), gallbladder and extrahepatic bile ducts (156), bronchus and lung (162.2–162.9), breast (174), prostate (185), kidney (189.0), and lymphatic and hematopoietic tissue (200–208). The follow-up interval was calculated from the date of baseline examination to the date of death or 31 December 2008, whichever occurred first. The median follow-up time among participants who did not develop cancer was 17.2 years.

We conducted and have presented the analyses for all cancers, except for those which had too few deaths to properly analyze (brain, skin, and reproductive organs). Analyses for HbA1c and HOMA-IR are only shown for cancers that we found a statistically significant association with diabetes or a suggestive trend for a cancer previously associated with diabetes such as colorectal cancer. For the other cancers, we confirmed that none of them was associated with HbA1c or HOMA-IR (data not shown).

### Statistical methods

Statistical analyses were conducted with Stata version 12.1 (StataCorp LP, College Station, Texas). The figures were conducted with R version 3.0.1 (R project for statistical computing). The prospective association between baseline diabetes status (as a dichotomous trait, as well as a categorical trait (i.e., no IFG, IFG, or diabetes)) and cancer mortality (overall and site specific) was assessed using Cox proportional hazards models with age as timescale and individual starting follow-up times treated as staggered entries. The nonparametric underlying baseline hazards were allowed to differ by study region using the strata option. By adding study region as strata, the model adjusts for region, but no association is estimated for region and region does not need to meet the assumption of proportionality of hazards. Initial Cox proportional hazards models accounted for age, sex, and region (model 1). Model 2 also adjusted for BMI, and in the case of breast cancer for baseline menopausal status (pre/post), hormone replacement therapy (current/past/never use), and parity (0/1–2/3–4/≥5). Model 3 further adjusted for education, smoking (never, former, current), and drinking status (never drinker, former drinker, light current drinker (<4 drinks/week), moderate current drinkers (4–12 drinks/week), heavy drinkers (>12 drinks/week)) [22]. The assumption of hazards proportionality was evaluated visually based on the smoothed association between age and scaled Schoenfeld residuals, with no major departures from proportionality.

To evaluate the relationship between HbA1c and cancer mortality, we modeled HbA1c as a continuous variable and derived adjusted hazard ratios of cancer mortality comparing the 80th versus the 20th percentiles of HbA1c. Additionally, HbA1c levels were separately modeled using restricted cubic splines with knots at the 10th (4.6 %), 50th (5.6 %), and 90th (10.7 %) percentile of its distribution.

HOMA-IR values were log-transformed, and their association with cancer mortality was evaluated using Cox proportional hazards models, using the same levels of adjustment as in models 1–3 but restricted to participants without diabetes. Plasma insulin was also log-transformed and analyzed similarly in a separate model.

Subgroup analyses (e.g., by sex, age groups, or smoking status) were not performed due to limited sample size, especially for specific cancer types. We performed several sensitivity analyses. First, we further adjusted for cigarette pack-years as well as for arsenic in the subset of participants with information on pack-years and urine arsenic concentrations available (*n* = 3,737). Arsenic is an established carcinogen that has been related to cancer mortality [23] and diabetes [24] in the Strong Heart Study. Second, we further adjusted for time since the diagnosis of diabetes. Third, to account for competing risks by causes of death other than cancer, we estimated proportional hazards regression models according to the method of Fine and Gray [25]. Findings from all sensitivity analyses were consistent with those reported (data not shown).

## Results

Tables 1 and 2 show participant characteristics stratified by cancer mortality and diabetes status, respectively. In this cohort of American Indians, there were 13.7 % participants with IFG and 45.7 % with diabetes at baseline. The prevalence of diabetes in this cohort was previously reported as 43 % for men and 52 % for women; a random sample (*n* = 311) of non-participants from this population showed a similar proportion of participants and non-participants with diabetes (40 and 38 %, respectively) [14]. During follow-up, 187 men and 243 women died from cancer, mainly from lung and prostate cancer in men and lung and breast cancer in women.

**Table 1:** Baseline characteristics of study participants overall and by cancer mortality status

	Overall (*n* = 4,419)	Cancer deaths (*n* = 430)	Non-cancer deaths or alive (*n* = 3,989)	*p* value*
Age (years)	55.1 (8.1)	60.4 (8.2)	54.6 (8.0)	<0.01
Men (%)	40.6	43.5	40.3	0.21
Arizona (%)	33.0	26.7	33.7	<0.01
Oklahoma (%)	32.4	32.6	33.4	0.71
North/South Dakota (%)	33.6	40.7	32.9	<0.01
<High school (%)	47.7	54.4	46.9	<0.01
Current smoking (%)	33.8	44.4	32.7	<0.01
Former smoking (%)	33.9	31.4	34.1	0.25
Current drinking (%)	41.4	35.1	42.1	<0.01
Obesity (BMI ≥ 30) (%)	50.9	46.3	51.3	0.05
Diabetes (%)**	45.7	44.9	45.8	0.73
Impaired fasting glucose (%)***	13.7	15.6	13.5	0.24
HOMA-IR (%)^a^	4.0 (3.6)	3.8 (3.5)	4.1 (3.6)	0.28
HbA1C (%)^b^	6.7 (2.4)	6.4 (2.1)	6.7 (2.5)	0.02

Data in the table are percentages for categorical variables or means (standard deviations) for continuous variables

* Based on the Chi-square test for qualitative variables and analysis of the variance for quantitative variables

** Defined as a fasting plasma glucose level ≥126 mg/dL

*** Defined as a fasting plasma glucose level of 110–125 mg/dL

^a^Based on 2,400 participants without diabetes and with HOMA-IR available

^b^Based on 4,116 participants with this information available

**Table 2:** Population characteristics by diabetes status

	Normal fasting glucose (*n* = 1,795)	Impaired fasting glucose (*n* = 606)*	Diabetes (*n* = 2,018)**	*p* value***
Age (years)	53.7 (8.0)	55.3 (8.4)	56.1 (7.9)	0.14
Men (%)	43.9	42.1	37.3	<0.01
Arizona (%)	19.2	26.9	47.2	<0.01
Oklahoma (%)	38.9	36.1	27.6	<0.01
North/South Dakota (%)	41.9	37.0	25.2	<0.01
<High school (%)	41.2	45.5	54.1	<0.01
Current smoking (%)	41.8	34.7	26.5	<0.01
Former smoking (%)	29.9	33.3	37.6	<0.01
Current drinking (%)	48.3	43.4	34.7	<0.01
Obesity (BMI ≥ 30) (%)	38.8	58.3	59.4	<0.01
HOMA-IR (%)^a^	3.4 (2.9)	6.0 (4.5)	–	<0.01
HbA1C (%)^b^	5.1 (0.6)	5.4 (0.7)	8.6 (2.4)	<0.01

Data in the table are percentages (standard errors) for categorical variables or means (standard errors) for continuous variables

* Defined as a fasting plasma glucose level of 110–125 mg/dL

** Defined as a fasting plasma glucose level ≥126 mg/dL

*** Based on the Chi-square test for qualitative variables and analysis of the variance for quantitative variables

^a^Based on 2,400 participants without diabetes and with HOMA-IR available

^b^Based on 4,116 participants with this information available

While confidence intervals are wide, due to small sample size, after multivariate adjustment for age, sex, BMI, education, drinking status, and smoking status (Table 3), the hazard ratio (95 % CI) for overall cancer mortality was 1.19 (0.97–1.45) for those with diabetes compared to those without. The corresponding hazard ratios (95 % CI) for gastric, liver, and prostate cancers were 4.09 (1.42–11.79), 2.94 (1.17–7.40), and 3.11 (1.22–7.94), respectively. Similar results were observed in models that further adjusted for log-transformed urine arsenic and for number of pack-years (*n* = 3,737), although the effect for overall cancer became significant in that analysis (HR 1.27; 95 % CI 1.03–1.58), and a suggestive, but not statistically significant, association was observed for colorectal cancer (HR 1.92; 95 % CI 0.92–4.00) (data not shown in tables). In the analysis of breast cancer, additional adjustment for parity and menopausal status did not affect our results. Similarly, adjustment for physical activity did not reveal significant changes in the risk of specific cancers, compared with model 3.

**Table 3:** Hazard ratios (95 % confidence interval) for all-cause and site-specific cancer mortality by diabetes status (*n* = 4,419). Bolded values are statistically significant

Type of cancer	Cases/non-cases	Model 1	Model 2	Model 3
Total cancer (*ICD-9 codes 140–208*)	428/3,969	1.14 (0.94–1.39)	1.16 (0.95–1.41)	1.20 (0.98–1.47)
Esophagus (*ICD 9 code* 150)	9/4,388	0.56 (0.13–2.33)	0.61 (0.14–2.65)	0.65 (0.15–2.82)
Stomach (*ICD 9 code 151*)	19/4,378	**4**.**00** (**1**.**42**–**11**.**30**)	**3**.**90** (**1**.**37**–**11**.**09**)	**4**.**20** (**1**.**45**–**12**.**13**)
Colon and rectum (*ICD 9 codes 153*–*154*)	35/4,397	1.72 (0.87–3.37)	1.65 (0.83–3.28)	1.77 (0.88–3.54)
Liver, intrahepatic bile ducts (*ICD 9 code 155*)	24/4,373	**2**.**72** (**1**.**10**–**6**.**76**)	**2**.**71** (**1**.**08**–**6**.**76**)	**2**.**93** (**1**.**16**–**7**.**38**)
Gallbladder, extrahepatic bile ducts (*ICD 9 code 156*)	13/4,384	0.91 (0.29–2.93)	0.79 (0.25–2.51)	0.63 (0.19–2.05)
Pancreas (*ICD 9 code 157*)	27/4,397	1.26 (0.58–2.76)	1.33 (0.60–2.93)	1.44 (0.65–3.21)
Trachea, bronchus, and lung (*ICD 9 code 162*)	83/4,314	0.62 (0.38–1.02)	0.70 (0.42–1.16)	0.72 (0.43–1.21)
Breast (*ICD 9 code 174*)^a^	26/2,587	1.41 (0.64–2.12)	1.38 (0.62–2.08)	1.32 (0.58–2.99)
Prostate (*ICD 9 code 185*)	20/1,764	2.37 (0.98–5.75)	**2**.**70** (**1**.**09**–**6**.**65**)	**2**.**96** (**1**.**15**–**7**.**57**)
Kidney (*ICD 9 code 189*)	30/4,367	0.96 (0.46–2.02)	0.91 (0.43–1.92)	0.91 (0.43–1.92)
Lymphatic and hematopoietic tissue (*ICD 9 codes 200*–*208*)	*43*/*4*,*376*	0.89 (0.48–1.66)	0.86 (0.46–1.61)	1.20 (0.98–1.47)

Model 1: adjusted for age and sex, stratified by center

Model 2: further adjusted for body mass index (<25; ≥25 and <30; ≥25 kg/m^2^)

Model 3: further adjusted for education, drinking status (never drinker/former drinker/light current drinker (<4 drinks/week)/moderate current drinkers (4–12 drinks/week)/heavy drinkers (>12 drinks/week)) and smoking status (never, former, current)

^a^Model 2 for breast cancer further adjusts for menopausal status, and models 3 and 4 further adjust for reproductive factors: menopause (yes/no) and parity (0, 1–2, 3–4, ≥5)

**Fig. 1:**
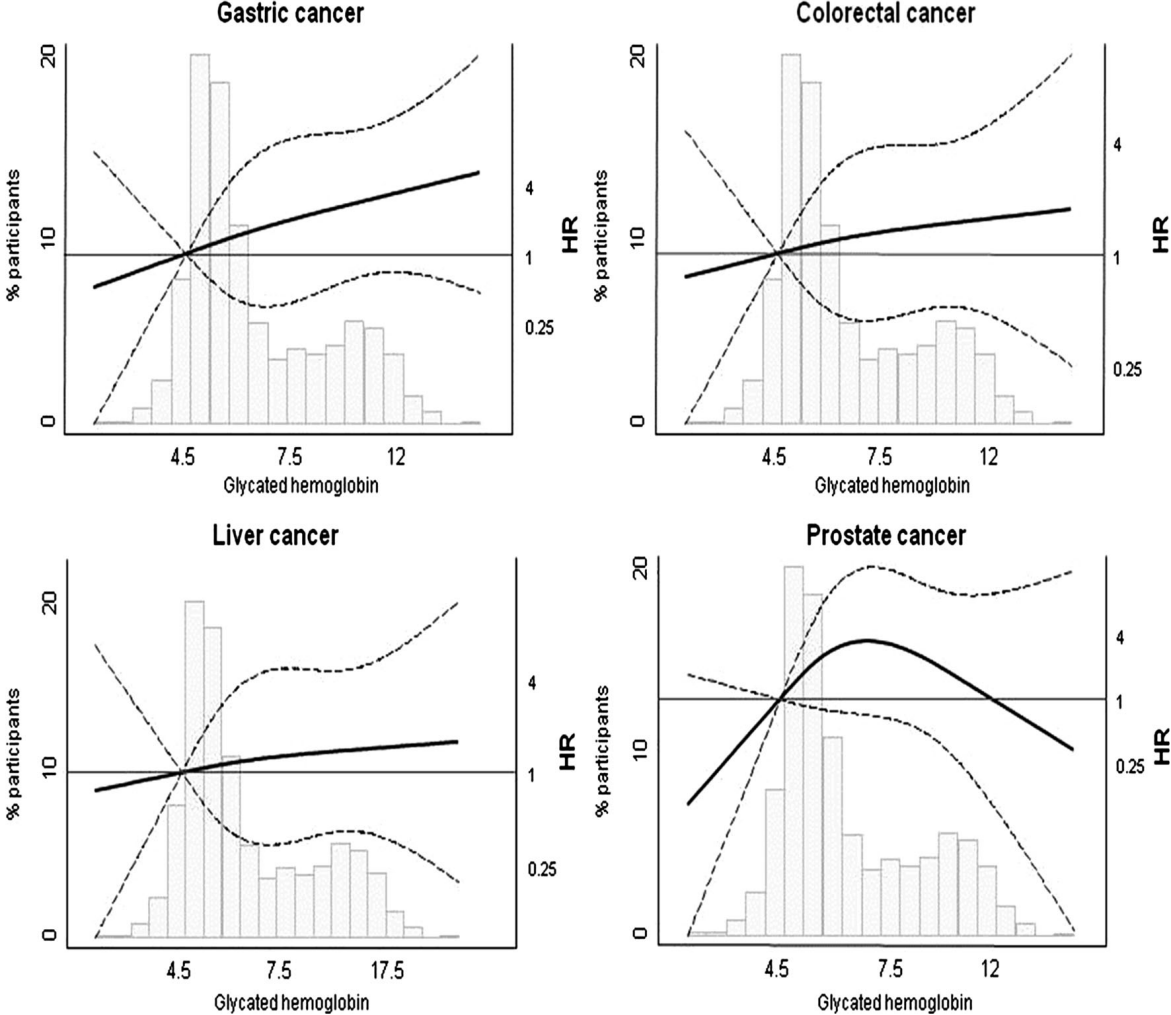
Distribution of glycated hemoglobin (% HbA1c) and hazard ratios for selected cancer mortality by % HbA1c

When modeling the dose–response relationship between HbA1C and cancer mortality, none of the associations were statistically significant (Fig. 1). For gastric cancer, the nonsignificant association was positive and fairly linear. For prostate cancer, the nonsignificant association was nonlinear. Total cancer was not associated with HbA1C (HR per IQR in HbA1c levels: 0.99; 95 % CI 0.85, 1.15).

Results for HOMA-IR and cancer (Table 4) showed only a positive association between HOMA-IR values and liver cancer mortality (HR 4.70 per log unit; 95 % CI 1.55–14.26). Unexpectedly, we also observed a negative association between HOMA-IR values and prostate cancer mortality. Similar findings were observed with plasma insulin (Table 4). HOMA-IR was not associated with total cancer mortality (HR per log unit of HOMA-IR was 0.92; 95 % CI 0.73, 1.18).

**Table 4:** Hazard ratios for site-specific cancer mortality among those without diabetes by insulin resistance (log HOMA-IR model) and log plasma insulin (*n* = 2,400).  Bolded values are statistically significant

Cancer	Cases/non-cases		HOMA-IR HR (95 % CI)	Plasma insulin HR (95 % CI)
Stomach	5/2,395	Model 1	0.64 (0.16–2.54)	1.03 (0.52–2.06)
		Model 2	0.33 (0.06–1.91)	0.94 (0.42–2.08)
		Model 3	0.29 (0.04–2.11)	0.95 (0.42–2.13)
Liver, intrahepatic bile ducts	7/2,393	Model 1	**4**.**68** (**1**.**57**–**13**.**98**)	**2**.**51** (**1**.**54**–**4**.**11**)
		Model 2	**4**.**72** (**1**.**47**–**15**.**13**)	**2**.**70** (**1**.**62**–**2**.**26**)
		Model 3	**4**.**70** (**1**.**55**–**14**.**26**)	**2**.**71** (**1**.**62**–**3**.**29**)
Prostate	9/2,391	Model 1	**0**.**34** (**0**.**13**–**0**.**83**)	**0**.**42** (**0**.**18**–**0**.**97**)
		Model 2	**0**.**23** (**0**.**07**–**0**.**75**)	0.51 (0.22–1.18)
		Model 3	0.25 (0.07–0.88)	0.82 (0.24–2.00)
Colon and rectum	35/2,384	Model 1	0.94 (0.44–1.99)	1.27 (0.79–2.03)
		Model 2	0.76 (0.31–1.92)	1.17 (0.68–2.03)
		Model 3	0.81 (0.32–2.08)	1.22 (0.71–2.11)

Model 1: adjusted for age and sex, stratified by center

Model 2: further adjusted for body mass index (<25; ≥25 and <30; ≥25 kg/m^2^)

Model 3: further adjusted for education, drinking status (never, former, current), smoking status (never, former, current)

## Discussion

In this population-based cohort study with almost 20 years of follow-up, we found that baseline type 2 diabetes was associated with a borderline significant increase risk of overall cancer mortality among American Indians from Arizona, Oklahoma, and North/South Dakota. The association became significant in a subset of the population with information available on arsenic exposure, after adjustment for this variable. Hyperglycemia, as measured by HbA1c, was also nonsignificantly associated with increased cancer risk. We found no clear association between baseline insulin resistance and overall cancer mortality, although HOMA-IR levels were positively associated with liver cancer and inversely associated with prostate cancer mortality.

The present study provides further evidence for increased risk of specific cancer mortality, including gastric and liver cancer, with baseline diabetes, although cautious interpretation is warranted, given the relatively small sample size. These findings are consistent with our a priori hypothesis based on reports conducted in Australia, East Asia, North America, and Europe [26–30]. Also consistent with our a priori hypothesis, we found a nonsignificant, but increased mortality risk estimate for colorectal cancer.

Hyperglycemia may contribute to the initial mutagenic events initiating neoplasia, via an increase in reactive oxygen species [31]. Additionally, two subsequent independent mechanisms enhancing carcinogenesis could promote cancer development: (1) the increased availability of glucose coupled with the Warburg effect and (2) the availability of endogenous insulin and insulin-like growth factors (IGFs), which are increased before and during the early course of type II diabetes and have a synergistic effect on tumor growth. Interestingly, Laron dwarfism, a syndrome characterized by a lack of functional IGF-1 receptors, confers a marked reduction in cancer risk [32]. In epidemiologic studies, treatment with insulin [33, 34] or insulin secretagogues [35] has been associated with an increased risk of some cancers, including breast, colon, pancreas, and prostate. Also, agents that decrease insulin resistance and thus decrease insulin levels (metformin) have been associated with a decreased risk of stomach, liver, pancreatic, and colorectal cancers [36], although there is recent conflicting evidence [37]. It is unclear whether hyperglycemia could show effects for certain, specific cancers and not for others.

In our study, HbA1c, HOMA-IR, and plasma insulin per se were associated with an increased risk of liver cancer mortality among those without diabetes, a finding that is consistent with previous epidemiologic studies [38–40].

An extensive review of meta-analyses points out various weaknesses in previous studies associating specific cancers with diabetes, but finds the most robust evidence for breast, intrahepatic cholangiocarcinoma, colorectal, and endometrial cancers [41]. This is consistent with our findings, while allowing for potential overlap in the classification of hepatocellular versus cholangiocarcinoma. Interestingly, the Beckwith–Wiedemann syndrome, with known elevations of insulin and IGFs during the first years of life, is associated with very high incidence of cancer in childhood, especially hepatoblastoma and Wilm’s tumor [42]. Another mechanism that may increase liver cancer risk of those with diabetes is the impaired immune function that potentially interferes with neoplastic surveillance and encourages chronic viral infection, such as hepatitis B and hepatitis C.

The association between diabetes and gastric cancer mortality in our study is consistent with findings from several meta-analyses published between 2011 and 2013 showing an increased risk of gastric cancer in individuals with diabetes. A recent review has considered the multiple interacting factors that may play a role in this association [43]. These include possibly higher infection rates or prevalence of Helicobacter pylori infection, potentially increased salt intake, or increased detection rates among those with diabetes requiring routine medical care for a chronic condition.

There appear to be differential effects of elevated levels of glucose and insulin during the prodromal/early stages of diabetes on the development of prostate cancer, compared with the influence of fully developed diabetes on the risk of prostate cancer mortality [44]. Evidence from a meta-analysis [45] and two large cohort studies [46, 47] showed lower prostate cancer incidence in individuals with long-standing diabetes compared to those without diabetes. The present study found that increased levels of insulin and insulin resistance (typical of subclinical diabetes) among those without diabetes were associated with lower risk of prostate cancer death. After the development of prostate cancer, however, the presence of diabetes seems to confer an increased risk of prostate cancer death. A recently published meta-analysis found an increased risk of prostate cancer mortality among those with diabetes [48]; however, a large collaborative study including a total of 2,217 prostate cancer deaths [49] found a nonsignificant decreased risk of mortality in individuals with diabetes. The present study provides further evidence for diabetes as a risk factor for prostate cancer death. Overall, the relationship of diabetes and its physiologic changes with prostate cancer incidence and mortality is complex [50, 51]. Future research is needed to more accurately consider the time course of these physiologic changes, as well as the social and medical influences on the relationship between prostate cancer development and associated mortality.

Interpretation of this epidemiologic evidence should be undertaken with caution considering the multiple and possibly countervailing influences of factors difficult to track over the relevant exposure period. These could include the direct effects of hyperglycemia as well as secondary physiologic responses to hyperglycemia (e.g., insulin and the effects of the insulin-like growth factor system) and treatment of diabetes. Although we adjusted for obesity, there could also be effects of obesity per se. The evaluation of the role of obesity on cancer is very complex, especially in a study that evaluates cancer mortality instead of cancer incidence. Indeed, at baseline, participants who later died of cancer were less likely to be obese compared to those without a subsequent cancer death. It is well known that the obesity of those with diabetes tends to ameliorate with advancing age; insulin levels decline with time as beta-cell function fails [52, 53]. There is also evidence that the influence of the IGF system declines with age in a parallel fashion to that of insulin [54]. It is challenging to confidently model or adjust for these multiple factors and their possible interactions. There are a number of effects, by which diabetes could increase the likelihood of death among those who have already developed cancer, such as increased cardiovascular complications and increased susceptibility to infections.

The Strong Heart Study was initially designed to study the prevalence of known or suspected cardiovascular disease risk factors in American Indians and to assess their influence on cardiovascular morbidity and mortality (http://strongheart.ouhsc.edu/). However, the long follow-up of Strong Heart Study participants has allowed us to secondarily evaluate the influence of some of these risk factors on cancer mortality. For this reason, we do not have information on certain confounders that are of obvious interest (e.g., family history of the studied cancers, stage at diagnosis, treatment, and some environmental exposures), making residual confounding likely. It was not possible to evaluate for potential confounding due to aspirin or non-steroidal anti-inflammatory agents because the only information available was on sporadic use. Since the analysis is based on cancer mortality, conclusions may have also been biased by differences in healthcare utilization, or access to more specialized services by those with diabetes, compared to those without diabetes. Finally, we lack incidence data, and particularly among tumors with high survival (e.g., breast or colorectal), the power to detect significant associations is limited by the small number of deaths from those cancers.

The strengths of this study include the prospective analysis, the availability of relevant covariates (including arsenic, an established carcinogen that was present in drinking water at levels above 10 µg/L, the current US EPA standard) [55] to adjust for confounding, and a population with a unique genetic background.

In a population with a high prevalence of diabetes and obesity and with unique environmental exposures, our data provide additional evidence of heightened risk of cancer mortality associated with diabetes and confirmation of increased risk of mortality due to stomach and liver cancers. Additional research is needed to evaluate the role of diabetes and obesity on cancer incidence in American Indian communities as well as the importance of diabetes prevention and control in reducing the burden of cancer incidence and mortality in the study population.
